# Taxonomic landscape of the *Mycobacterium* “*Fortuitum-Vaccae*” clade: a genome study

**DOI:** 10.3389/fmicb.2026.1728622

**Published:** 2026-02-16

**Authors:** Chengcheng Wang, Yu Feng, Dan Zhou, Feifei Zhao, Yuling Xiao, Yi Xie, Alan McNally, Zhiyong Zong

**Affiliations:** 1 Center of Infectious Diseases, West China Hospital, Sichuan University, Chengdu, China; 2 Division of Infectious Diseases, State Key Laboratory of Biotherapy, Chengdu, China; 3 Laboratory of Pathogen Research, West China Hospital, Sichuan University, Chengdu, China; 4 Department of Laboratory Medicine, West China Hospital, Sichuan University, Chengdu, China; 5 Department of Microbes, Infection and Microbiomes, School of Infection, Inflammation and Immunology, College of Medicine and Health, University of Birmingham, Birmingham, United Kingdom; 6 State Key Laboratory of Respiratory Health and Multimorbidity, Chengdu, China

**Keywords:** *Mycobacterium*, *Mycobacterium fortuitum*, *Mycobacterium vaccae*, non-tuberculosis mycobacteria, taxonomy

## Abstract

**Introduction:**

The *Mycobacterium* “*Fortuitum-Vaccae*” clade (FVC) contains many rapid-growing species and is increasingly reported in human infections globally. However, the clade’s taxonomic composition and the exact association with human infections remain to be explored.

**Methods:**

In this study, we conducted a comprehensive genome-based analysis of the FVC, aiming to update its taxonomy and identify new taxa. We compiled a dataset of 298 *Mycobacterium* species using public databases, ultimately including 222 type strain genomes for phylogenomic analysis

**Results:**

This revealed 106 species within the FVC. Subsequent curation of 11,534 public *Mycobacterium* identified 557 belonging to the FVC, from which we uncovered 86 new taxa (genomospecies) and resolved one new synonym pair. We reconstructed three core-protein phylogenomic trees and found the FVC comprising eight distinct subclades. Our analysis unveiled a remarkably complicated taxonomic landscape within the clade, encompassing 106 known species, among which 16 species, such as *M. syngnathidarum* and *M. yunnanensis*, have not previously been classified within the FVC. We detected a pair of synonyms: *M. murale* is a heterotypic synonym of *M. tokaiense*.

**Discussion:**

These findings highlight the remarkable taxonomic diversity within the FVC. The updated taxonomy and the curated database of genomes allow precise species identification in diagnostics, clinical reporting, epidemiological surveillance, and future studies on the ecology and pathogenesis. Accurate identification may uncover the disease spectrum, clinical manifestation, and prognosis of infections by individual FVC species, guiding countermeasures.

## 1 Introduction

Non-tuberculous mycobacteria constitute a diverse group of over 200 identified species to date (LPSN, 2022). They are often found in environmental niches, including soil, dust, and water (Johansen et al., 2020) but also well known to be opportunistic pathogens (Dahl et al., 2022). Despite their prevalence and emerging pathogenic potential (Varghese and Al-Hajoj, 2020), a comprehensive understanding of their taxonomy and pathogenicity is lacking.

The “*Fortuitum-Vaccae*” clade (FVC), named after its prominent members *Mycobacterium fortuitum* and *Mycobacterium vaccae*, have historically been recognized as a large group of rapidly growing mycobacteria other than species in the “*Abscessus-Chelonae*” clade (Gupta et al., 2018). The FVC has a complicated taxonomy encompassing over 100 species. Some species of the FVC have been found to be synonyms; for instance, *Mycobacterium vanbaalenii* and *Mycobacterium conceptionense* are a synonym of *Mycobacterium austroafricanum* and *Mycobacterium senegalense*, respectively (Pan et al., 2022). In addition, novel species that are likely of the FVC (considering the highest average nucleotide identity [ANI] of the type strains with those of known species within this clade) are commonly reported (Cheng et al., 2021). We adopted an operational definition in this study: comparing to the cluster formed by the “*Abscessus-Chelonae*” clade, FVC encompasses all *Mycobacterium* species in the cluster containing *M. fortuitum* and *M. vaccae* in our core-protein phylogenies. The cluster of FVC may comprise multiple branches in the phylogenies, representing subclades (groups).

A study published in 2018 (Gupta et al., 2018) transferred species of the FVC from the genus *Mycobacterium* to a new genus named *Mycolicibacterium*. Subsequently, the new species names with *Mycolicibacterium* have been included in a validation list of International Journal of Systematic and Evolutionary Microbiology (Oren and Garrity, 2018), the official publication of the International Committee on Systematics of Prokaryotes. However, it has also been proposed to keep using the original *Mycobacterium* names, given that the new nomenclature has the potential to cause confusion and provides no benefits to the field of clinical mycobacteriology (Tortoli et al., 2019a; Meehan et al., 2021). The original names with *Mycobacterium* continue to be validly published (Tortoli, 2019). We consistently used the species names with *Mycobacterium* in this study and hereafter we applied *M.* for all *Mycobacterium* species names for brevity.

The pathogenic potential of the FVC has historically been underestimated, characterized by previous perceptions of it being primarily environmental rather than pathogenic (Gupta et al., 2018). The large number of species within the FVC is fascinating in taxonomic perspectives but poses great challenges for accurate species identification and causes confusion in clinical practice. Clinical reports and surveillance studies based on incorrect species identification could lead to wrong information and mislead countermeasures. Accurate species identification is crucial for diagnosis, surveillance, and elucidating pathogenicity and allows to identify some species or strains with enhanced virulence and antimicrobial resistance (Adekambi and Drancourt, 2004; Mediavilla-Gradolph et al., 2015).

To address this significant knowledge gap, we conducted a comprehensive genome-based analysis of the taxonomic landscape of the FVC including all known *Mycobacterium* species. This allowed us to precisely define the FVC, curate and update its taxonomy, and uncover its species composition. We also applied the updated taxonomy to curate all available genomes of the FVC in National Center for Biotechnology Information (NCBI). We therefore further identified 86 previously unknown taxa (genomospecies) belonging to the FVC and constructed a database comprising all genomes of these clade.

## 2 Methods

### 2.1 Collecting *Mycobacterium* species dataset

We examined all known *Mycobacterium* species listed in List of Prokaryotic Names with Standing in Nomenclature (LPSN; https://lpsn.dsmz.de; *n* = 287) and NCBI (https://www.ncbi.nlm.nih.gov; *n* = 262) (accessed on 30 June 2023 for both). LPSN was used exclusively as a reference for valid species names (to compile the list of *Mycobacterium* species), not for genome retrieval. Genomes were sourced from NCBI. After manual deduplication when merging those in LPSN and NCBI together, there are a total of 298 *Mycobacterium* species (Supplementary Dataset S1). We conducted thorough searches for type strain genomes in NCBI (including both assembly and Sequence Read Archive [SRA] datasets). Species without a designated type strain or without an available genome sequence of the type strain were excluded. For *Mycobacterium mucogenicum*, as no assembled genome of the type strain (JCM 13575) was available, we identified SRA data for this strain. We downloaded and assembled these data *de novo* into contigs using SPAdes-based assembly pipeline Shovill v1.1.0^1^ to include this type strain in our comparative genomic analysis. We excluded 75 species from further analysis due to: unavailability of the type strain genome in NCBI (*n* = 10), unavailability of genome assemblies in NCBI (*n* = 40), being actual subspecies rather than species (*n* = 6), being members of *Mycobacterium tuberculosis* (*n* = 5), having misspelled species names (*n* = 5), being heterotypic (*n* = 3) or homotypic (*n* = 2) synonyms, having been transferred to another genus (*n* = 2), and uncultured candidatus species (*n* = 2). Then, we retrieved the type strain genome sequence of all included *Mycobacterium* species from NCBI (*n* = 223, as of June 30, 2023; Supplementary Dataset S1). Notably, the only available genome of *Mycobacterium aurantiacum* in NCBI was contaminated. As such, we constructed a dataset comprising the remaining 222 *Mycobacterium* species.

### 2.2 Integration of phylogenomic and genomic similarity metrics

Our analysis began with phylogenomic inference, which provided the evolutionary context necessary to generate species hypotheses. We first reconstructed three core-protein phylogenomic trees encompassing all 222 *Mycobacterium* species, which revealed the overall phylogenetic structure of the genus. These trees also served as the primary guide for identifying candidate species clusters: species that grouped closely in the phylogeny were flagged as potential conspecifics or close relatives, warranting further investigation of their genomic similarity. The ANI and digital DNA–DNA hybridization (dDDH) thresholds were used to validate the species hypotheses generated by phylogenomics. These methods were used complementarily, with phylogenomics forming the foundational framework and ANI/dDDH serving as quantitative validation of species boundaries.

### 2.3 Quality control of genome sequences

We checked genome completeness and contamination using CheckM v1.0.18 (Parks et al., 2015) and then annotated genomes using Prokka v1.14.5 (Seemann, 2014). We discarded genome assemblies of low quality defined as consisting of >500 contigs, having <90% genome completeness, or >10% genome contamination (detailed in Supplementary Dataset S1), ensuring high-quality genomic data.

### 2.4 Initial species assignment using fastANI v1.32

We calculated pairwise ANI using fastANI v1.32 (Jain et al., 2018) and used a ≥95% ANI cutoff (Dahl et al., 2021; Pan et al., 2022a) to compare each genome with type strains of known *Mycobacterium* species: (1) Genomes with ≥95% ANI to one single type strain were tentatively assigned to that species. (2) Genomes with ≥95% ANI to two or more type strains were flagged as ambiguous, triggering a dDDH validation step. (3) Genomes with <95% ANI to all known type strains were classified as “unassigned to known species.

### 2.5 dDDH validation for ambiguous assignments

For genomes with conflicting ANI results (assigned to more than one species using ≥95% ANI alone), we applied a more stringent criterion of ≥95% ANI plus ≥70% dDDH (Richter and Rossello-Mora, 2009; Meier-Kolthoff et al., 2013) (1) genomes met the dual threshold for one single type strain and were definitively assigned to that species; (2) genomes showed ≥95% ANI but <70% dDDH with two or more type species. This conflict was resolved by classifying it as “species undetermined,” which may represent a new species or part of a broader species complex need to be determined.

### 2.6 Identification of novel taxa (genomospecies)

Genomes that could not be assigned to any known species (i.e., they had an ANI of <95% to all type strains) were considered potential novel taxa. To confirm their phylogenetic position within the FVC, we inferred a phylogenomic tree including these genomes and all type strains. A group of genomes forming a distinct, well-supported clade and sharing ≥95% ANI among themselves was defined as a new genomospecies.

### 2.7 Synonym definition

Species synonyms were defined if they clustered tightly in the phylogenomic tree and exceeded the ANI/dDDH thresholds: ≥95% ANI (Dahl et al., 2021; Pan et al., 2022) plus ≥70% dDDH (Richter and Rossello-Mora, 2009; Meier-Kolthoff et al., 2013).

### 2.8 Phylogenomic analysis of *Mycobacterium* species and the “*Fortuitum-Vaccae*” clade

**Figure 1:**
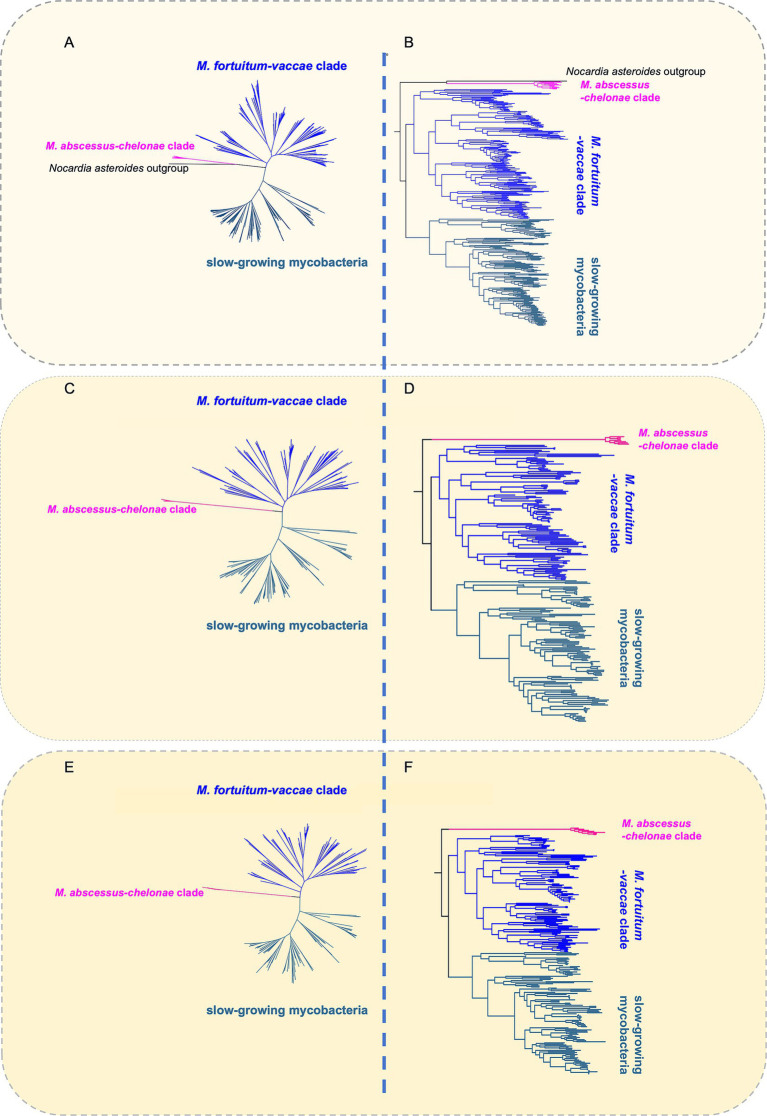
Phylogenomic trees of species within the *genus Mycobacterium.* Phylogenomic trees for 222 *Mycobacterium* species were constructed using three distinct sets of conserved genetic markers: The first set relied on conserved genes identified via GTDB-Tk; the second utilized 1,862 core protein families identified through the CD-HIT program; and the third was based on 136 conserved proteins that constitute the marker set for the phylum *Actinobacteria*. **(A,C,E)** The resulting phylogenomic trees distinctly segregates the rapidly and slowly growing *Mycobacterium* species into two major branches. **(B,D,F)** The “*Abscessus-Chelonae*” clade and the “*Fortuitum-Vaccae*” clade (FVC) are the clade found in fast-growing *Mycobacterium*, but they show no signs as a monophyletic group. The “*Abscessus-Chelonae*” clade comprises a distinct monophyletic clade, setting itself apart from all other *Mycobacterium* species with a deep branch that is thought to be the most ancestral within the genus (Tortoli et al., 2017). By contrast, FVC forms a monophyletic lineage with slow-growing *Mycobacterium*.

Phylogenomic trees were constructed for 222 *Mycobacterium* species using three distinct sets of conserved genetic markers (Figure 1). The first phylogenetic tree was based on concatenated amino acid sequences encoded by conserved genes-these genes were identified and aligned using GTDB-Tk v2.3.2 (Chaumeil et al., 2022) with default settings, and constructed using IQ-TREE v2.3.0 (Minh et al., 2020) under LG model allowing for sites heterogeneity with 1,000 ultrafast bootstraps. For the second tree, 1,862 core protein families were identified using the CD-HIT program as described in Gupta et al. (2018); leveraging these core proteins, the tree was also built with IQ-TREE v2.3.0 (Minh et al., 2020). The third comprehensive phylogenomic tree was constructed from concatenated sequences for 136 proteins, as detailed in Gupta et al. (2018), which form the established marker set for the phylum *Actinobacteria*.

**Figure 2:**
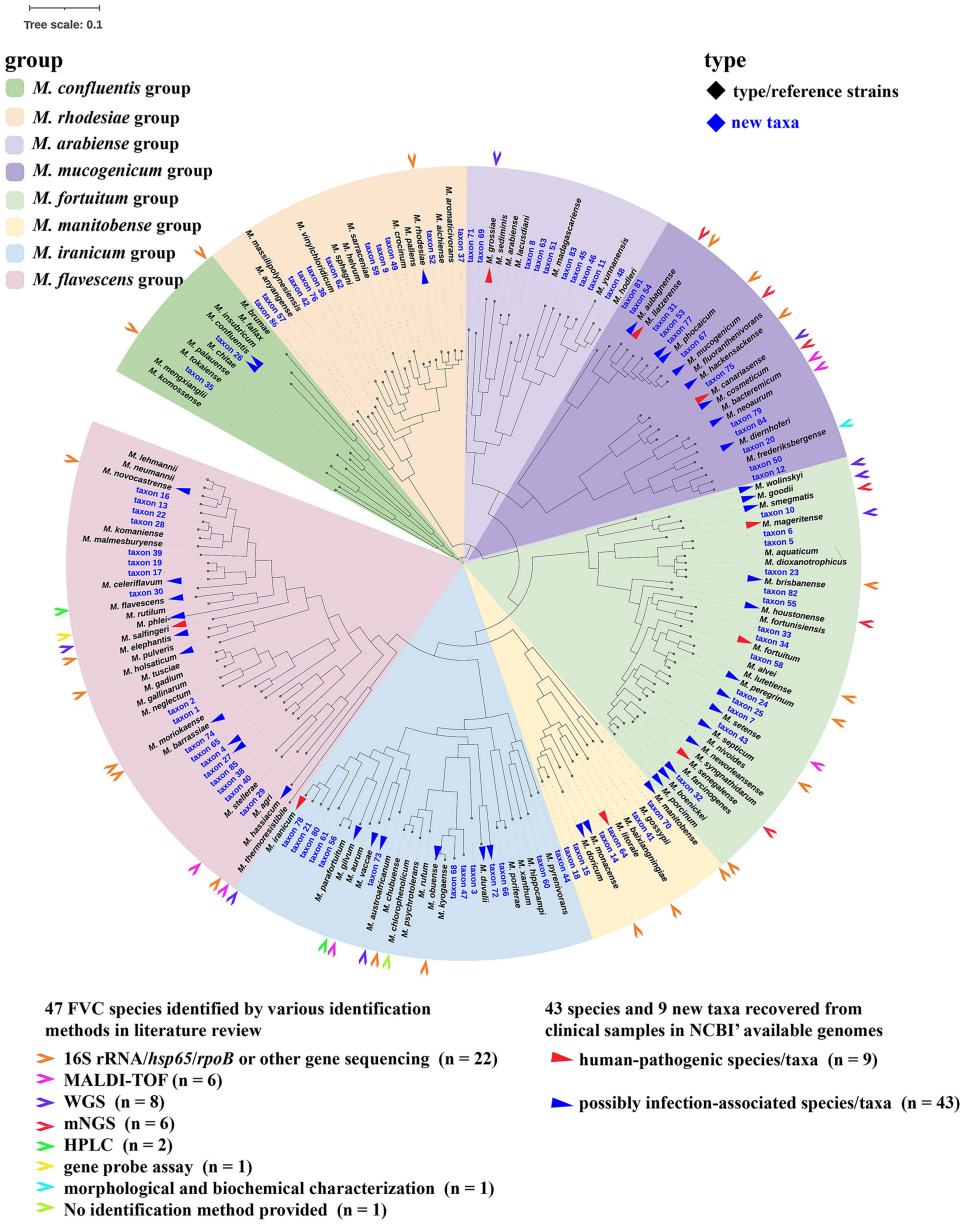
A phylogenomic tree of 106 type strains and 86 novel taxa within the *Mycobacterium* “*Fortuitum-Vaccae*” clade. One hundred and twenty marker genes of 106 type strains (Table 1) and reference strains of 86 novel taxa (Supplementary Table S2) were identified and aligned using GTDB-Tk v2.3.2 (Chaumeil et al., 2022) with default settings. A phylogenomic tree based on the concatenated protein sequences encoded by marker genes was then inferred using IQ-TREE v2.3.0 (Minh et al., 2020) under LG model allowing for sites heterogeneity with 1,000 ultra-fast bootstraps and was visualized and annotated using iTOL v6.9 (Letunic and Bork, 2021). Bar, value indicates the nucleotide substitutions per site. The FVC comprised eight major subclades with 11 to 42 species/taxa in each subclade.

Based on the phylogenomic tree of type strains of all *Mycobacterium* species (Figure 1), we further selected those (*n* = 106) belonging to the FVC to infer three FVC-specific phylogenomic trees with abovementioned methods (Figure 2 and Supplementary Figure S1). All trees were visualized and annotated using iTOL v6.9 (Letunic and Bork, 2021).

### 2.9 Curation of species identification for the “*Fortuitum-Vaccae*” genomes in GenBank

We used txid1762 [Organism:exp] AND “latest” [filter] to search NCBI GenBank, and then retrieved all available assemblies (*n* = 11,534, accessed on 30 June 2023) of *Mycobacterium*. We discarded genomes labelled ‘atypical’ in NCBI for the following reasons: (i) derived from metagenome, (ii) contaminated, (iii) with many frameshifted proteins, (iv) fragmented assembly, (v) too large or too small genome length, (vi) low quality sequence, or (vii) missing rRNA or tRNA genes. Detailed explanations of atypical genomes are available in the NCBI’s Genome Notes.^2^ We then evaluated genomes for the quality of assemblies using QUAST v5.0.2 (Mikheenko et al., 2018) and checked for genome completeness and contamination using CheckM v1.0.18 (Parks et al., 2015). We further discarded genome assemblies of low quality defined as consisting of >500 contigs, having <90% genome completeness, or >10% genome contamination. We calculated ANI and dDDH values between each of the genomes and type strains of *Mycobacterium* genomes as described above.

### 2.10 Software and data availability

All software was used with default parameters unless otherwise specified. Key software versions include: GTDB-Tk v2.3.2 for phylogenomics, IQ-TREE v2.3.0 for tree inference, CheckM v1.0.18 for quality assessment, fastANI v1.32 for ANI calculations, and the Genome-to-Genome Distance Calculator (GGDC) for dDDH estimates. All genomes were retrieved from NCBI database. The complete list of the 222 type strain genomes with NCBI accession numbers is provided in Supplementary Dataset S1. The list of 11,534 public *Mycobacterium* genomes analyzed, their quality metrics, and their final species assignments are provided in Supplementary Dataset S2. The list of 86 new genomospecies and their representative genomes is provided in Supplementary Table S2.

## 3 Results

### 3.1 The “*Fortuitum-Vaccae*” clade of *Mycobacterium* comprises 106 defined species

We constructed a dataset comprising 222 *Mycobacterium* species and the genome sequence of their type strains sourced from NCBI. Unlike previous investigations which have had a narrower spectrum of 110–150 species (Tortoli et al., 2017; Gupta et al., 2018; Nouioui et al., 2018b; Bachmann et al., 2020), our dataset encompasses 222 *Mycobacterium* species. From the retrieved genomes we inferred three independent core-protein phylogenomic trees comprising all 222 *Mycobacterium* species (Figure 1), to ensure the robustness and consistency of our phylogenetic conclusions, as using different marker sets helps mitigate potential biases inherent to any single method.

The phylogenomic trees segregates *Mycobacterium* species into three super clades, namely the “*Abscessus-Chelonae*” clade, the FVC, and the slow-growing *Mycobacterium* (Figure 1). The “*Abscessus-Chelonae*” clade forms a separate monophyletic clade, distinguishing itself from all other *Mycobacterium* species with a deep branch (Figure 1), indicative of it being the ancestral *Mycobacterium* species, as previously reported (Tortoli et al., 2017). The FVC shares a common ancestor with slow-growing *Mycobacterium*. Based on the phylogenomic trees, we were able to define the FVC. We identified 106 species within the FVC, 16 (such as *M. syngnathidarum* and *M. yunnanensis*) of which have not been previously classified in the FVC (Nouioui et al., 2018b) (Table 1). Among the 107 species, 106 are published (Table 1). We found three slow-growing species, namely *Mycobacterium doricum* (Tortoli et al., 2001), *Mycobacterium salfingeri* (Musser et al., 2022), and *Mycobacterium tuscia* (Tortoli et al., 1999), within the predominantly fast-growing FVC. The unexpected coexistence of slow-growing species within the predominantly fast-growing clade may suggest complex evolutionary adaptations that require further mechanistic investigation (Bachmann et al., 2020; Zhu et al., 2023). Unlike the monophyletic “*Abscessus-Chelonae*” clade, FVC comprised eight well-supported subclades (assigned Group 1 to 8 here) with between 6 and 23 species in each group (Figure 2, Supplementary Figure S1, and Supplementary Table S3).

**Table 1:** Updated classification and nomenclature of *Mycobacterium* species within the “*Fortuitum-Vaccae*” clade.

Species name^a^	Type or reference strain	Genome accession no.	Original proposer and year
Species (*n* = 106)^b^			
*M. agri*	CCUG 37673^T^	PDCP00000000	Tsukamura (1981)
*M. aichiense*	NCTC 10820^T^	UGQK00000000	Tsukamura et al. (1981)
*M. alvei*	JCM 12272^T^	AP022565	Ausina et al. (1992)
*M. anyangense*	JCM 30275^T^	AP022620	Kim et al. (2015)
*M. arabiense*	JCM 18538^T^	AP022593	Zhang et al. (2013)
*M. aromaticivorans*	JCM 16368^T^	JALN00000000	Hennessee et al. (2009)
*M. aubagnense*	DSM 45150^T^	POTN00000000	Adekambi et al. (2006)
*M. aurum*	NCTC 10437^T^	CVQQ00000000	Tsukamura (1966a)
*M. austroafricanum*	DSM 44191^T^	HG964450	Tsukamura et al. (1983c)
*M. bacteremicum*	DSM 45578^T^	MVHJ00000000	Brown-Elliott et al. (2010)
*M. baixiangningiae*	LJ126^T^	CP066218	Cheng et al. (2021)
*M. boenickei*	JCM 15653^T^	AP022579	Schinsky et al. (2004)
*M. brisbanense*	JCM 15654^T^	BCSX00000000	Schinsky et al. (2004)
*M. brumae*	CIP 1034565^T^	PDCN02000000	Luquin et al. (1993)
*M. canariasense*	CCUG 47953^T^	LQOL00000000	Soledad Jimenez et al. (2004)
*M. celeriflavum*	DSM 46765^T^	MVHN00000000	Shahraki et al. (2015)
*M. chitae*	NCTC 10485^T^	LR134355	Tsukamura (1967)
*M. chlorophenolicum*	JCM 7439^T^	BCQY00000000	Apajalahti et al. (1986)
*M. chubuense*	DSM 44219^T^	MVHO00000000	Tsukamura et al. (1981)
*M. confluentis*	DSM 44017^T^	LQOQ00000000	Kirschner et al. (1992)
*M. cosmeticum*	DSM 44829^T^	CCBB000000000	Cooksey et al. (2004)
*M. crocinum*	JCM 16369^T^	BBHD00000000	Hennessee et al. (2009)
*M. diernhoferi*	ATCC 19340^T^	CP080332	Tsukamura et al. (1983c)
*M. doricum*	DSM 44339^T^	LQOS00000000	Tortoli et al. (2001)
*M. duvalii*	JCM 6396^T^	AP022563	Stanford and Gunthorpe (1971)
*M. elephantis*	DSM 44368^T^	JACKTZ000000000	Shojaei et al. (2000)
*M. fallax*	DSM 44179^T^	LQOJ00000000	Levy-Frebault et al. (1983)
*M. farcinogenes*	DSM 43637^T^	CCAY000000000^i^	Chamoiseau (1973)
*M. flavescens*	DSM 43991^T^	JACKUL000000000	Bojalil et al. (1962)
*M. fluoranthenivorans*	DSM 44556^T^	JAANOW000000000	Hormisch et al. (2004)
*M. fortuitum*	JCM 6387^T^	AP025518	Da Costa Cruz (1938)
*M. frederiksbergense*	DSM 45364^T^	JACKTH000000000	Willumsen et al. (2001)
*M. gadium*	JCM 12688^T^	AP022608	Casal and Calero (1974)
*M. gilvum*	NCTC 10742^T^	UGQM00000000	Stanford and Gunthorpe (1971)
*M. goodii*	ATCC 700504^T^	CP092364	Brown et al. (1999)
*M. gossypii*	S2-37^T^	JAFEVR010000015	Huang et al. (2021)
*M. hassiacum*	DSM 44199^T^	KB903840	Schroder et al. (1997)
*M. helvum*	JCM 30396^T^	AP022596	Tran and Dahl (2016)
*M. hippocampi*	DSM 45391^T^	JACKSE000000000	Balcazar et al. (2014)
*M. hodleri*	JCM 12141^T^	BBGO00000000	Kleespies et al. (1996)
*M. holsaticum*	JCM 12374^T^	CP080998	Richter et al. (2002)
*M. houstonense*	ATCC 49403^T^	FJVO00000000	Schinsky et al. (2004)
*M. insubricum*	DSM 45132^T^	MVHS00000000	Tortoli et al. (2009)
*M. iranicum*	DSM 45541^T^	LQPC00000000	Shojaei et al. (2013)
*M. komaniense*	GPK 1020^T^	CVTA00000000	Gcebe et al. (2018)
*M. komossense*	DSM 44078^T^	JACKTY000000000	Kazda and Muller (1979)
*M. lacusdiani*	JXJ CY 35^T^	JAKCFC000000000	Xiao et al. (2022)
*M. litorale*	JCM 17423^T^	AP022586	Zhang et al. (2012)
*M. llatzerense*	MG13^T^	LXOV00000000	Gomila et al. (2008)
*M. lutetiense*	DSM 46713^T^	JAGIOP000000000	Konjek et al. (2016)
*M. madagascariense*	JCM 13574^T^	AP022610	Kazda et al. (1992)
*M. mageritense*	CIP 104973^T^	CCBF000000000	Domenech et al. (1997)
*M. malmesburyense*	WCM 7299^T^	CVTB00000000	Gcebe et al. (2017)
*M. mengxianglii*	Z-34^T^	CP065373	Cheng et al. (2021)
*M. monacense*	DSM 44395^T^	MVIA00000000	Reischl et al. (2006)
*M. moriokaense*	JCM 6375^T^	AP022560	Tsukamura et al. (1986)
*M. mucogenicum*	JCM 13575^Td^	DRX263051	Springer et al. (1995)
*M. neoaurum*	ATCC 25795^T^	JMDW00000000	Tsukamura (1972)
*M. neworleansense*	ATCC 49404^T^	CWKH00000000	Schinsky et al. (2004)
*M. nivoides*	DL90^T^	CP034072	Dahl et al. (2021)
*M. novocastrense*	JCM 18114^T^	BCTA00000000	Shojaei et al. (1997)
*M. obuense*	DSM 44075^T^	JYNU00000000	Tsukamura et al. (1981)
*M. pallens*	JCM 16370^T^	BBHE00000000	Hennessee et al. (2009)
*M. parafortuitum*	CCUG 20999^T^	MVID00000000	Tsukamura (1966b)
*M. peregrinum*	DSM 43271^T^	LQPP00000000	Kusunoki and Ezaki (1992)
*M. phlei*	CCUG 21000^T^	CP014475	Lehmann et al. (1899)
*M. phocaicum*	DSM 45104^T^	POTM00000000	Adekambi et al. (2006)
*M. porcinum*	DSM 44242^T^	JACKVC000000000	Tsukamura et al. (1983b)
*M. poriferae*	JCM 12603^T^	AP022570	Padgitt and Moshier (1987)
*M. psychrotolerans*	JCM 13323^T^	AP022574	Trujillo et al. (2004)
*M. pulveris*	JCM 6370^T^	AP022599	Tsukamura et al. (1983a)
*M. pyrenivorans*	JCM 15927^T^	BBHB00000000	Derz et al. (2004)
*M. rhodesiae*	DSM 44223^T^	MVIH00000000	Tsukamura et al. (1981)
*M. rufum*	JCM 16372^T^	BBGS00000000	Hennessee et al. (2009)
*M. rutilum*	JCM 16371^T^	BBHF00000000	Hennessee et al. (2009)
*M. sarraceniae*	JCM 30395^T^	AP022595	Tran and Dahl (2016)
*M. sediminis*	DSM 45643^T^	JACKUW000000000	Zhang et al. (2013)
*M. senegalense*	ATCC 35796^T^	CP081000	Chamoiseau (1973)
*M. septicum*	DSM 44393^T^	CBMO000000000	Schinsky et al. (2000)
*M. setense*	DSM 45070^T^	JTJW00000000	Lamy et al. (2008)
*M. smegmatis*	NCTC 8159^T^	LN831039	Lehmann et al. (1899)
*M. sphagni*	ATCC 33027^T^	NOZR00000000	Kazda (1980)
*M. stellerae*	CECT 8783^T^	RARC00000000	Nouioui et al. (2019)
*M. thermoresistibile*	ATCC 19527^T^	AGVE00000000	Tsukamura (1966a)
*M. tokaiense*	NCTC10821^T^	UGQT00000000	Tsukamura et al. (1981)
*M. tusciae*	DSM 44338^T^	MVIM00000000	Tortoli et al. (1999)
*M. vaccae*	ATCC 25954^T^	JH814683	Bonicke and Juhasz (1964)
*M. vinylchloridicum*	CECT 8761^T^	JACBJQ000000000	Cortes-Albayay et al. (2021)
*M. wolinskyi*	ATCC 700010^T^	LQQA00000000	Brown et al. (1999)
*M. xanthum*	Y57^T^	JAIFZS000000000	Pan et al. (2022)
*M. aquaticum* ^c^	RW6^T^	MVHF00000000	Hashemi Shahraki et al. (2017)
*M. barrassiae* ^c^	CCUG 50398^T^	JACKUK010000001	Adékambi et al. (2006)
*M. dioxanotrophicus* ^c^	PH-06^T^	CP020809	He et al. (2017)
*M. fortunisiensis* ^c^	TNTM28^T^	VOMB01000005	Gharbi et al. (2021)
*M. grossiae* ^c^	DSM 104744^T^	CP043474	Paniz-Mondolfi et al. (2017)
*M. hackensackense* ^c^	DSM 44833^T^	JACKUC010000001	Hong et al. (2003)
*M. kyogaense* ^c^	NCTC 11659^T^	QJUA00000000	Nouioui et al. (2018a)
*M. lehmannii* ^c^	CECT 8763^T^	NKCN00000000	Nouioui et al. (2017)
*M. manitobense* ^c^	DSM 44615^T^	JACKSJ010000001	Turenne et al. (2003)
*M. massilipolynesiensis* ^c^	M26^T^	LN929900	Phelippeau et al. (2017)
*M. neglectum* ^c^	CECT 8778^T^	NVQE00000000	Nouioui et al. (2018c)
*M. neumannii* ^c^	CECT 8766^T^	NKCO00000000	Nouioui et al. (2017)
*M. palauense* ^c^	CECT 8779^T^	NVQF00000000	Nouioui et al. (2018c)
*M. salfingeri* ^c^	20-157661^T^	CP081006	Musser et al. (2022)
*M. syngnathidarum* ^c^	27335^T^	MLCL00000000	Fogelson et al. (2018)
*M. yunnanensis* ^c^	DSM 44838^T^	JACKVK010000001	Li et al.^1^
Species rejected (*n* = 4)			
*M. vanbaalenii* ^e^	PYR-1^T^	*M. austroafricanum*	
*M. conceptionense* ^f^	CCUG 50187^T^	*M. senegalense*	
*M. murale* ^g^	JCM 13392^T^	*M. tokaiense*	
Species listed in LPSN but moved out of FVC (*n* = 1)			
*M. vulneris* ^h^	DSM 45247^T^	NCXM01000000	

a Gupta et al. (2018) to transfer species of the FVC from the genus *Mycobacterium* to a new genus named *Mycolicibacterium.* The new species names with *Mycolicibacterium* have been included in a validation list by IJSEM (Oren and Garrity, 2018). However, Tortoli et al. (2019a) and Meehan et al. (2021) proposed to keep using the original names with *Mycobacterium*, given that the new nomenclature has the potential to cause confusion and provides no benefits to the field of clinical mycobacteriology. The original names remain validly published, and anyone is free to use them (Tortoli, 2019). In our study, we use the original names.

b The species in bold are validly published and those underlined are effectively published, while those neither in bold nor underlined have not been validly or effectively published.

c *M. aquaticum, M. barrassiae, M. dioxanotrophicus, M. fortunisiensis, M. grossiae, M. hackensackense, M. kyogaense, M. lehmannii, M. manitobense, M. massilipolynesiensis, M. neglectum, M. neumannii, M. palauense, M. salfingeri, M. syngnathidarum,* and *M. yunnanensis* have not been included in the study by Gupta et al. (2018).

^C^ For *M. mucogenicum*, while no assembled genome of the type strain (JCM 13575) was available, we identified SRA data for this strain. We downloaded and assembled these data *de novo* into contigs using Shovill v1.1.0 (https://github.com/tseemann/shovill) to include this type strain in our comparative genomic analysis.

e *M. vanbaalenii* is proposed to be a later heterotypic synonym of *M. austroafricanum* (Tortoli et al., 2019b).

f *M. conceptionense* is proposed to be a later heterotypic synonym of *M. senegalense* (Tortoli et al., 2019b).

g *M. murale* is proposed to be a later heterotypic synonym of *M. tokaiense* based on genome analysis in this study.

h Tortoli et al. (2019a) and Gupta (2019) have reported that the type strain of *Mycolicibacterium vulneris*, DSM 45247^T^ (accession no. NCXM01000000) is clusterd within the slow-growing group of mycobacteria, and proposed that *Mycolicibacterium vulneris* (Gupta et al., 2018) should be reinstated to its previous basonym *Mycobacterium vulneris* (van Ingen et al., 2009) as a slow grower. Therefore, *M. vulneris* labled as “*Mycolicibacterium*” in LPSN is not included in this table.

i It has been pointed out previously (Turenne, 2019) that the genome of *Mycobacterium farcinogenes* type strain DSM 43637^T^ (accession no. CCAY000000000) is actually obtained from a strain of *Mycobacterium senegalense.*

1 https://www.ncbi.nlm.nih.gov/Taxonomy/Browser/wwwtax.cgi?id=368477.

We examined all 107 species within the FVC to identify possible synonyms that may not have been previously recognized to perform species curation. Notably, the genome labeled *M. farcinogenes* type strain DSM 43637^T^ (accession no. CCAY000000000) is actually sequenced from a strain of *M. senegalense* (Turenne, 2019). The genuine genome sequence of *M. farcinogenes* type strain is not available yet; hence, we did not include this species for identifying synonyms. We employed a combined approach of phylogenomic tree inference and ANI/dDDH. We applied a stringent criterion of a ≥ 95% ANI plus a ≥ 70% dDDH value to define synonyms. As such, we detected a pair of synonyms.

*Mycobacterium murale* (Vuorio et al., 1999) is a heterotypic synonym of *Mycobacterium tokaiense* (Tsukamura et al., 1981). After detailed analysis of *M. tokaiense* and *M. murale* from their original articles (Tsukamura et al., 1981; Vuorio et al., 1999), the phenotypic and genotypic features of the emended *M. tokaiense* are as follows. Phenotypically, as for cell morphology and staining, it consists of Gram-stain-positive, acid-fast bacilli. These bacilli exhibit both strong acid-fastness and weak or partial acid-fastness. The cells can take the form of long rods, often exceeding 7 μm in length. Regarding colony characteristics, colonies are typically smooth and may be cream-colored and non-pigmented as previously described for *M. tokaiense*. However, it has been observed that in certain cases, similar to what is seen in *M. murale*, the colonies may be pigmented. For growth characteristics, the organism grows within a temperature range of 10–37 °C. At 10 °C, growth is variable, with the majority showing positive results within 10 days, while growth at 45 °C is generally weak within 5 days. In the phylogenomic tree, *M. murale* JCM 13392^T^ and *M. tokaiense* NCTC 10821^T^ cluster together. Genotypically, the draft genome sequences of *M. murale* JCM 13392^T^ (accession no. BLKT00000000) and *M. tokaiense* NCTC 10821^T^ (accession no. UGQT00000000) have an dDDH value of 83.8% and an ANI value of 97.98%. Both the ANI and dDDH analyses indicate that the two species are the same species. Based on principles in the International Code of Nomenclature of Prokaryotes (ICNP) (2022 Revision) (Oren et al., 2023), specifically Rule 24b, *M. tokaiense* has the priority of the species name over *M. murale*.

We emended description of *Mycobacterium tokaiense* Tsukamura et al. 1981. *Mycobacterium tokaiense* was originally described by Tsukamura et al. 1981. Based on the evidence presented here, this species description is emended by Wang et al. in 2025 to include the phenotypic and genotypic characteristics of the previously described *Mycobacterium murale* Vuorio et al. 1999, which is now considered a later heterotypic synonym of *M. tokaiense*. The emended species should be cited as: *Mycobacterium tokaiense* Tsukamura et al. 1981 emended by Wang et al. in 2025.

*Mycobacterium tokaiense* Tsukamura et al. 1981

= *Mycobacterium murale* Vuorio et al. 1999.

The type strain is 47503 (previously, strain 5553) = ATCC 27282 = CIP 106807 = DSM 44635 = JCM 6373 = NCTC 10821.

Hence, we refined the total count of 107 initially identified species to 106 species, encompassing 105 published and one unpublished (Table 1).

We also found 10 species of five pairs with close evolutionary relatedness-clustering together within the phylogenomic tree of the FVC (Figure 2), but conflicting genomic similarity metrics: *Mycobacterium obuense*/*Mycobacterium kyogaense*, *Mycobacterium fluoranthenivorans*/*Mycobacterium hackensackense*, *Mycobacterium chubuense*/*Mycobacterium chlorophenolicum*, *Mycobacterium neumannii*/*Mycobacterium lehmannii*, and *Mycobacterium septicum*/*Mycobacterium nivoides* (Supplementary Table S1). These pairs shared a ≥ 95% ANI (range: 95.1–96.3%) but a < 70% dDDH value (<70%; range: 60.9–68.1%), failing to meet the stringent criterion that we used for defining synonyms but are nonetheless closely related and warrant further studies to clarify their taxonomic positions.

### 3.2 Curation of genomes in the FVC with the updated taxonomy leads to identification of 86 new taxa (genomospecies)

We applied our updated FVC taxonomy to curate publicly available genomes of the FVC in GenBank (accessed on 30 June 2023). Considering that strains labelled “*Mycolicibacterium*” in NCBI do not fully represent all FVC, we retrieved all *Mycobacterium* assemblies (*n* = 11,534, accessed by 30 June 2023) from GenBank. We excluded genomes (*n* = 559) labelled ‘atypical’ in NCBI for the following reasons: derived from metagenome (*n* = 169); contaminated (*n* = 146); containing many frameshifted proteins (*n* = 113); fragmented assembly (*n* = 111); too large (*n* = 8) or too small (*n* = 4) genome length; low quality sequence (*n* = 3); missing rRNA genes (*n* = 1) or tRNA genes (*n* = 1); and not of *Mycobacterium* (*n* = 3). We further discarded an additional 180 assemblies due to low quality defined by >500 contigs (*n* = 169), <90% genome completeness (*n* = 8), or >10% genome contamination (*n* = 3).

**Figure 3:**
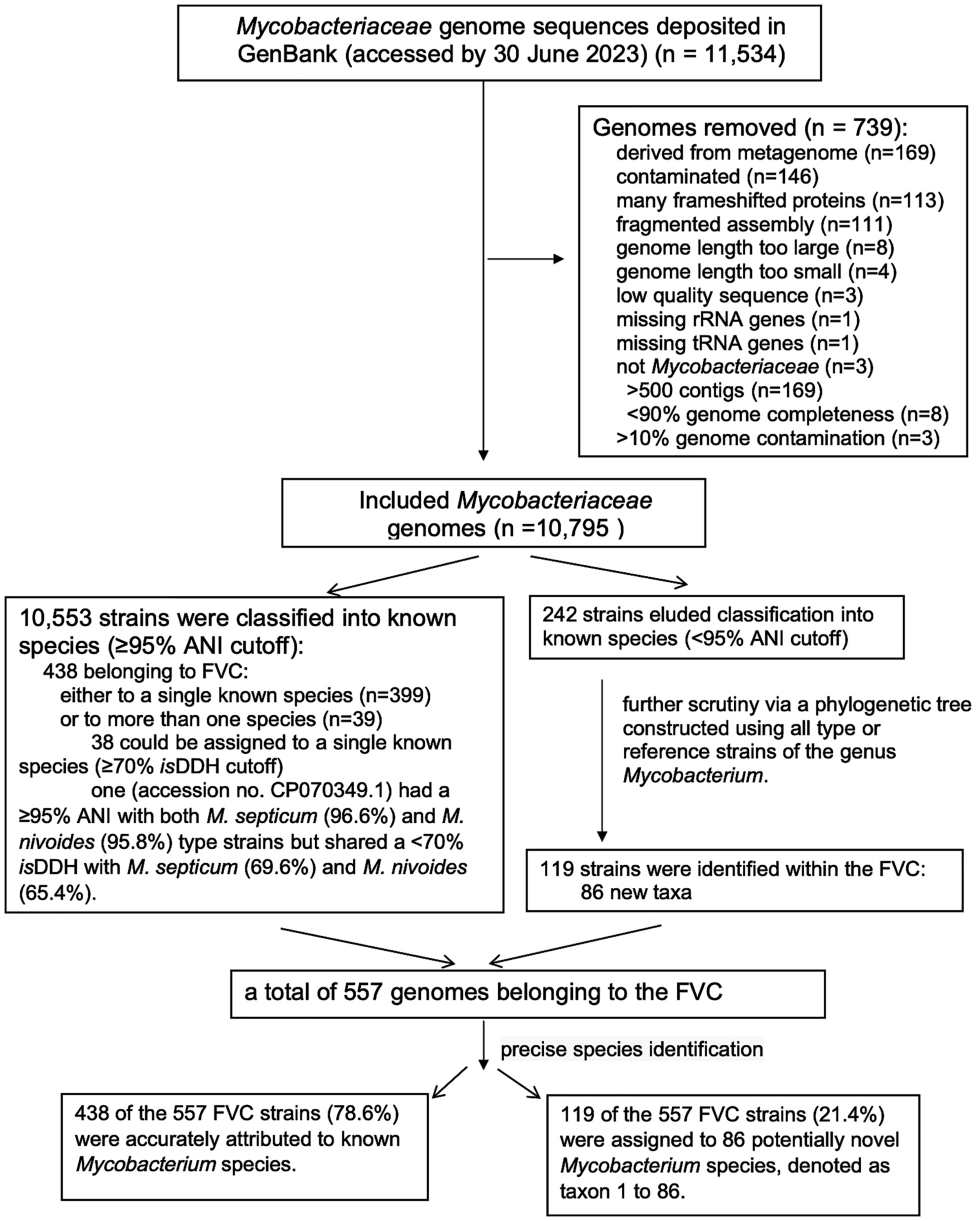
A schematic outline of methods and main results during curation of genomes in FVC.

We determined the precise species for the remaining 10,795 *Mycobacterium* genomes (Figure 3; Supplementary Dataset S2). Using a ≥ 95% ANI cutoff, 10,553 strains were assigned to at least one known species, with 438 belonging to FVC, either to a single known species (*n* = 399) or to more than one species (*n* = 39). For the 39 genomes assigned to more than one species, we further determined their dDDH values and applied a ≥ 70% cutoff for species identification. Of these 39 genomes, 38 could be assigned to a single known species. However, the remaining one (accession no. CP070349.1) had a ≥ 95% ANI with both *M. septicum* (96.6%) and *M. nivoides* (95.8%) type strains but shared a < 70% dDDH with *M. septicum* (69.6%) and *M. nivoides* (65.4%). Therefore, its species cannot be determined and may represent a new species or alternatively, this strain together with *M. septicum* and *M. nivoides* could represent a common species with pairwise ANI ≥ 95% (Supplementary Table S1). A total of 242 genomes had a < 95% ANI with all known species and could not be assigned to any known species. To determine their taxonomic position, we inferred a new phylogenomic tree comprising of these 242 genomes and the type or reference strains of all known mycobacterial species (Supplementary Figure S2). We found that 119 were located within the FVC and could be assigned to 86 new taxa (genomospecies) using a ≥ 95% ANI cutoff, denoted as taxon 1 to 86 (Figure 2; Supplementary Table S2). These 86 new taxa also belong to the eight abovementioned well-supported major groups (Figure 2). These new taxa could be considered naming as Candidatus *Mycobacterium fortuitum-vaccae* clade taxon 1 to 86. Further phenotypic characterization of the new taxa is required to establish their species status with proper names according to ICNP (2022 Revision) (Oren et al., 2023), specifically Rule 27 and Recommendation 30.

## 4 Discussion

In this study, we precisely defined the FVC and unveiled its remarkably complicated taxonomic landscape encompassing 106 known species, accounting for almost half of all *Mycobacterium* species. The remarkably large number of species within the FVC stands in strong contrast to the other rapid-growing “*Abscessus-Chelonae*” clade, which currently comprises only six species. This disparity may reflect a true difference in evolutionary diversification, but it should be interpreted cautiously, as it could also be influenced by a historical research bias towards characterizing environmental mycobacteria, leading to the FVC’s recent and rapid expansion. We also revealed that FVC comprising eight well-supported subclades. We then curated 11,534 *Mycobacterium* genomes and detected 557 belonging to the FVC. From these we uncovered 86 new taxa (genomospecies), which are very likely novel species, further highlighting the remarkable taxonomic diversity within FVC.

Our expanded dataset of 222 species allowed us to authoritatively assign 106 species to the FVC, including 16 species not previously classified within it. This refined taxonomy is not merely a taxonomic exercise; it provides a crucial, accurate framework for all subsequent studies on the evolution, ecology, and pathogenicity of this group. Such a large number of species within FVC raises the question of how species in the clade are divided. The mechanisms and factors driving the divergence of the FVC to shape the evolutionary trajectory and develop the remarkable species diversity have not been understood. Additionally, this analysis allowed for the systematic exploration of the FVC’s more intricate structure, highlighting its diversity with eight distinct major subclades.

Along with several FVC species identified recently (Cheng et al., 2021; Pan et al., 2022), the above findings highlight that the FVC is a highly diverse and complex group. By applying the updated taxonomy and stringent criteria (≥95% ANI and ≥70% dDDH) (Richter and Rossello-Mora, 2009; Meier-Kolthoff et al., 2013) to publicly available genomes, we identified 86 tentative novel taxa (genomospecies) within the FVC. These taxa, supported by ANI and phylogenomic analyses, highlight the significant underrepresentation of genomic diversity in previous studies (Gupta et al., 2018; Cheng et al., 2021; Meehan et al., 2021). Interestingly, the new taxa were distributed across all eight major groups, further underscoring the FVC’s taxonomic complexity and evolutionary breadth. The inclusion of these taxa in a curated database provides a valuable resource for future studies on the pathogenicity, ecology, and evolution of the FVC. However, the identification of new taxa necessitates further investigation on these tentative species using both genomic and phenotypic methods to establish their species status, as well as to propose appropriate species names in accordance with the current prokaryotic nomenclature code (Oren et al., 2023), specifically Rule 27 and Recommendation 30.

Additionally, we validated a new synonym pair, *M. murale* and *M. tokaiense*, based on genomic evidence, aligning with recent taxonomic revisions in *Mycobacterium* (Tortoli, 2019). This highlights the critical role of genomic approaches in clarifying species boundaries, particularly in groups with poorly defined taxonomies (Pan et al., 2022). However, our study also uncovered instances where pairs of species (e.g., *M. septicum* and *M. nivoides*) exhibited conflicting ANI and dDDH results, suggesting that these species are closely related but may require further investigation to resolve their taxonomic status definitively. This points to the limitations of relying solely on ANI and dDDH for species delineation and highlights the need for additional phenotypic and ecological data to complement genomic analyses (Konstantinidis and Tiedje, 2005).

The ecological diversity within the FVC, reflected by the presence of species with varying growth rates and habitat preferences, warrants further investigation. The FVC contains species that are capable of thriving in diverse environmental niches, including soil, water, and clinical settings (Johansen et al., 2020). This ecological versatility raises important questions about the evolutionary pressures driving species divergence within the clade. The presence of slow-growing species like *M. doricum* (Tortoli et al., 2001), *M. salfingeri* (Musser et al., 2022), and *M. tuscia* (Tortoli et al., 1999) within the FVC could result from historical misclassification or inaccurate phenotypic growth-rate estimation under differing laboratory conditions. Alternatively, it may reflect secondary adaptations to environmental niches selecting for slower replication, such as nutrient limitation or stress tolerance (Bachmann et al., 2020; Zhu et al., 2023). Further studies are needed to explore how these ecological factors shape the genetic and phenotypic diversity of the FVC, as well as their role in adaptation to diverse environmental conditions (Balcazar et al., 2014; Johansen et al., 2020; Gharbi et al., 2021).

Our curated taxonomy fills critical gaps in the FVC, with direct clinical utility. Conventional methods such as biochemical tests and 16S rRNA sequencing often misclassify closely related NTM species (Adekambi and Drancourt, 2004; Mediavilla-Gradolph et al., 2015). Considering the lowering price and widening availability of genome sequencing, we encourage using genome-based approaches (e.g., ANI comparison) for precise identification of FVC species. If genome-based identification is used, we propose to clearly describe the identification methods and state the possibility of misidentification as a limitation in any future published studies and reports. Matrix-assisted laser desorption/ionization-time-of-flight mass (MALDI-TOF MS) is a conventional method with increasing use in clinical microbiology. It will be helpful to improve species identification for the FVC by MALDI-TOF MS aligning with genome-based assignment. Incorrect species identification could mask some clinically relevant species with important antimicrobial resistance and enhance virulence, which require rigorous targeted surveillance, and prevent us from accurate understanding many critical aspects of pathogens such as the disease spectrum, clinical manifestation, prognosis, transmission, and prevalence, therefore hindering the design and implementation of countermeasures. FVC comprises clinically important pathogens causing difficult-to-treat infections. Precise species identification is truly needed to enhance our understanding and guide countermeasures.

While our study provides a comprehensive genomic overview of the FVC, certain limitations should be considered. First, we recognized that taxonomic delineations such as the 95% ANI or 70% dDDH thresholds represent human-imposed conventions applied to a continuum of natural genomic diversity, and these definitions may not fully capture the ecological or evolutionary complexity of prokaryotic populations. Second, the analysis was inherently constrained by the availability and quality of genomes in public databases. The representation of taxa was uneven, with clinically relevant or frequently isolated species having multiple genome sequences, while environmental or rare species may be represented by only a single type strain genome, if at all. This sampling bias could influence our perception of the clade’ true diversity and the robustness of the phylogenomic groups for less-represented species. Future efforts to sequence underrepresented taxa will be crucial to refine this taxonomic framework further. Third, we caution against over interpreting FVC species as ‘human-pathogenic’. Historical reports of FVC-related infections suffer from several limitations: (1) common misidentification prior to genome-based methods, e.g., confusing *M. fortuitum* with closely related species; (2) ambiguity between colonization and disease, especially in immunocompromised or cystic fibrosis patient, (3) variable quality of older literature such as lack of standardized case definitions, and (4) underreporting of environmental or non-clinical isolates. As such, unless pathogenicity is clearly proven, we use the term “infection-associated” to reflect documented isolation from clinical specimens, not definitive proof of pathogenicity. Furthermore, our study is focused on *in silico* methods, additional phenotypic characterization of the new taxa identified is needed to establish their species status in accordance with the current prokaryotic nomenclature code (Oren et al., 2023), specifically Rule 27 and Recommendation 30. Prioritize phenotypic characterization of novel taxa, particularly those within the large, clinically relevant clade encompassing the well-characterized *M. fortuitum,* by targeting antimicrobial susceptibility profiles, growth conditions, and chemotaxonomic markers (e.g., mycolic acid profiles) to complement genomic data. Additionally, we encourage the mycobacteriology community to leverage our curated taxonomy for reclassifying historical isolates and updating clinical databases (e.g., LPSN and NCBI Taxonomy), thereby ensuring consistent species delineation and reporting across research and clinical practice.

In conclusion, our comprehensive genomic analysis of the FVC provides significant taxonomic and phylogenomic insights, refining the clade’ species composition and uncovering previously unrecognized diversity. These findings not only address gaps in the taxonomy of FVC but also pave the way for future studies on the evolution, ecology, and pathogenicity of the FVC.

## Data Availability

The datasets presented in this study can be found in online repositories. The names of the repository/repositories and accession number(s) can be found in the article/Supplementary material.

## Footnotes

1
https://github.com/tseemann/shovill

2
https://www.ncbi.nlm.nih.gov/datasets/docs/v2/policies-annotation/genome-processing/genome_notes/
