# Supplementary material for: Taxonomic landscape of the Mycobacterium “Fortuitum-Vaccae” clade: a genome study
Source: Front Microbiol. 2026 Feb 16;17:1728622. doi: 10.3389/fmicb.2026.1728622 (PMC12950695; doi:10.3389/fmicb.2026.1728622)

Table S1. 10 species of five pairs sharing a ≥95% ANI but a <70% dDDH value

| Pairs | ANI (%) | dDDH (%) |
| --- | --- | --- |
| *M. obuense* and *M. kyogaense* | 96.3 | 68.1 |
| *M. fluoranthenivorans* and *M. hackensackense* | 96.1 | 63.4 |
| *M. chubuense* and *M. chlorophenolicum* | 95.8 | 65.0 |
| *M. neumannii* and *M. lehmannii* | 95.1 | 61.0 |
| *M. septicum* and *M. nivoides* | 95.1 | 60.9 |

Table S2**.** Tentative taxon assignations for novel, unnamed *Mycobacterium* species identified in this study.

| Taxon | Reference | Accession | Closest known species |  | Closest taxon | |
| --- | --- | --- | --- | --- | --- | --- |
|  | strain^a^ | no., GCA_ | species | ANI/*is*DDH  (%) | Taxon | ANI/dDDH  (%) |
| 1 | NBB3 | 000230895.3 | *M. gallinarum* | 84.8/27.2 | 2 | 84.4/29.6 |
| 2 | JS617 | 000243415.3 | *M. neglectum* | 89.6/38.7 | 1 | 84.3/28.9 |
| 3 | NBB4 | 000266905.1 | *M. chlorophenolicum* | 83.8/26.4 | 73 | 82.3/27.1 |
| 4 | JS623 | 000328565.1 | *M. rutilum* | 81.3/27.5 | 65 | 87.9/35.1 |
| 5 | 155 | 000373905.1 | *M. dioxanotrophicus* | 86.3/40.3 | 6 | 94.1/54.8 |
| 6 | 141 | 000382405.1 | *M. dioxanotrophicus* | 86.5/41.2 | 5 | 94.1/54.3 |
| 7 | URHD0025 | 000426065.1 | *M. setense* | 91.2/45.0 | 43 | 87.7/33.2 |
| 8 | URHB0044 | 000620625.1 | *M. arabiense* | 81.2/24.0 | 63 | 83.4/26.7 |
| 9 | EPa45 | 001021385.1 | *M. crocinum* | 88.1/35.2 | 49 | 88.9/35.4 |
| 10 | X7B | 001187505.1 | *M. smegmatis* | 88.8/35.7 | 32 | 82.0/28.3 |
| 11 | Root135 | 001426545.1 | *M. hodleri* | 88.4/34.7 | 46 | 93.4/46.7 |
| 12 | Root265 | 001428895.1 | *M. frederiksbergense* | 89.8/40.2 | 50 | 90.4/44.3 |
| 13 | GA-1285 | 001499905.1 | *M. novocastrense* | 89.7/39.8 | 16 | 89.2/39.5 |
| 14 | GA-2829 | 001499965.1 | *M. monacense* | 86.2/40.0 | 15 | 86.2/40.1 |
| 15 | IS-1496 | 001499995.1 | *M. monacense* | 89.4/37.3 | 18 | 93.1/52.4 |
| 16 | IS-1590 | 001500025.1 | *M. novocastrense* | 92.3/48.7 | 13 | 89.1/37.2 |
| 17 | IS-3022 | 001500065.1 | *M. celeriflavum* | 92.1/48.1 | 19 | 94.8/56.1 |
| 18 | IS-1742 | 001500125.1 | *M. monacense* | 89.1/37.6 | 15 | 93.1/53.2 |
| 19 | GA-1999 | 001500145.1 | *M. celeriflavum* | 92.0/45.0 | 17 | 94.9/55.1 |
| 20 | YC-RL4 | 001644575.1 | *M. frederiksbergense* | 87.2/31.5 | 12 | 86.9/28.9 |
| 21 | H39 | 001650495.1 | *M. iranicum* | 89.6/38.8 | 78 | 89.4/37.6 |
| 22 | 852002-51961_  SCH5331710 | 001665535.1 | *M. komanii* | 93.6/53.1 | 28 | 94.4/54.9 |
| 23 | 852013-50091_  SCH5140682 | 001665685.1 | *M. brisbanense* | 92.2/48.5 | 6 | 85.7/39.8 |
| 24 | 852002-51209_  SCH5440388 | 001665785.1 | *M. peregrinum* | 93.8/55.2 | 25 | 94.4/55.7 |
| 25 | ACS819 | 001667205.1 | *M. peregrinum* | 94.2/55.6 | 24 | 94.4/55.2 |
| 26 | ACS4331 | 001667265.1 | *M. confluentis* | 90.1/39.5 | 84 | 80.4/24.8 |
| 27 | ACS1612 | 001667505.1 | *M. rutilum* | 81.5/28.7 | 85 | 92.9/49.2 |
| 28 | 1164985.4 | 001668575.1 | *M. komanii* | 92.9/49.1 | 22 | 94.5/53.2 |
| 29 | 1274761 | 001668615.1 | *M. stellerae* | 82.4/25.0 | 40 | 82.6/25.3 |
| 30 | E740 | 001672895.1 | *M. celeriflavum* | 85.7/28.8 | 19 | 85.7/25.6 |
| 31 | ST-F2 | 001905655.1 | *M. phocaicum* | 90.3/39.0 | 67 | 90.1/38.7 |
| 32 | HMC1 | 001942045.1 | *M. boenickei* | 92.9/49.5 | 43 | 88.8/36.7 |
| 33 | E802 | 001666875.1 | *M. septicum* | 85.6/28.8 | 34 | 90.6/40.5 |
| 34 | GA-1841 | 001954135.1 | *M. septicum* | 85.6/28.7 | 33 | 90.5/40.2 |
| 35 | MS1601 | 001984215.1 | *M. tokaiense* | 84.6/27.5 | 41 | 79.9/25.8 |
| 36 | shizuoka-1 | 002723835.1 | *M. vinylchloridicum* | 85.0/29.5 | 59 | 85.2/29.3 |
| 37 | ENV421 | 002887815.1 | *M. aromaticivorans* | 93.7/66.1 | 52 | 91.8/57.2 |
| 38 | GAS496 | 003201655.1 | *M. rutilum* | 80.9/28.7 | 65 | 82.7/26.4 |
| 39 | GF69 | 003284965.1 | *M. novocastrense* | 85.7/29.4 | 19 | 85.1/28.1 |
| 40 | DSM 44221 | 003347205.1 | *M. stellerae* | 83.7/26.2 | 29 | 82.7/26.1 |
| 41 | PS03-16 | 004570325.1 | *M. litorale* | 84.4/27.3 | 64 | 84.2/27.1 |
| 42 | CH28 | 004745805.1 | *M. helvum* | 83.2/27.1 | 36 | 83.4/26.4 |
| 43 | DL99 | 004762045.1 | *M. nivoides* | 91.0/44.9 | 32 | 88.6/30.1 |
| 44 | CR10 | 005222675.1 | *M. pyrenivorans* | 84.2/26.3 | 60 | 83.3/26.4 |
| 45 | S5.20 | 006439015.1 | *M. hodleri* | 82.5/23.7 | 83 | 83.3/26.7 |
| 46 | AT1 | 002043095.1 | *M. hodleri* | 88.5/36.5 | 11 | 93.3/47.5 |
| 47 | 018/SC-01/001 | 007096635.1 | *M. kyogaense* | 87.1/31.4 | 68 | 87.1/35.7 |
| 48 | P1-18 | 008329535.1 | *M. hodleri* | 89.9/39.0 | 46 | 88.3/39.2 |
| 49 | P1-5 | 008329565.1 | *M. pallens* | 87.7/32.6 | 9 | 88.9/38.9 |
| 50 | P9-22 | 008329585.1 | *M. frederiksbergense* | 89.2/36.9 | 12 | 90.4/40.1 |
| 51 | P9-64 | 008329605.1 | *M. grossiae* | 80.8/23.3 | 63 | 88.5/37.4 |
| 52 | ELW1 | 008329905.1 | *M. aichiense* | 92.8/49.5 | 37 | 91.9/43.2 |
| 53 | CBMA226 | 009729075.1 | *M. mucogenicum* | 89.5/37.6 | 77 | 89.9/39.2 |
| 54 | CBMA311 | 009729085.1 | *M. llatzerense* | 86.5/30.5 | 31 | 86.3/35.4 |
| 55 | CBMA247 | 009729095.1 | *M. septicum* | 87.7/33.2 | 43 | 87.8/37.5 |
| 56 | JCM 6367 | 010725485.1 | *M. parafortuitum* | 94.5/54.7 | 73 | 83.4/32.1 |
| 57 | DL592 | 011694515.1 | *M. massilipolynesiensis* | 86.1/40.0 | 36 | 82.8/26.8 |
| 58 | DL440 | 011745145.1 | *M. lutetiense* | 89.2/37.6 | 25 | 89.1/30.1 |
| 59 | ENV482 | 013337765.1 | *M. helvum* | 88.7/36.5 | 62 | 86.9/27.5 |
| 60 | DL | 013390125.1 | *M. hippocampi* | 84.3/28.7 | 44 | 83.4/25.7 |
| 61 | AT2.18 | 014190915.1 | *M. austroafricanum* | 83.1/27.1 | 80 | 83.0/24.4 |
| 62 | BK086 | 004362315.1 | *M. vinylchloridicum* | 87.7/33.0 | 59 | 86.8/27.7 |
| 63 | AZCC_0083 | 014202335.1 | *M. arabiense* | 80.9/28.7 | 51 | 88.4/28.7 |
| 64 | NIIDNTM18 | 014218295.1 | *M. litorale* | 88.2/36.5 | 15 | 86.7/28.3 |
| 65 | OAS707 | 014873705.1 | *M. rutilum* | 81.3/28.7 | 4 | 87.7/29.4 |
| 66 | PO1 | 017312405.1 | *M. poriferae* | 94.5/55.8 | 73 | 81.7/24.7 |
| 67 | TY66 | 018326145.1 | *M. mucogenicum* | 93.3/51.2 | 77 | 91.2/32.6 |
| 68 | PSTR-4-N | 022348085.1 | *M. obuense* | 88.2/36.1 | 47 | 87.2/27.8 |
| 69 | YH-1 | 022557175.1 | *M. lacusdiani* | 81.4/28.7 | 8 | 80.8/24.0 |
| 70 | F2034L | 023015925.1 | *M. gossypii* | 87.1/33.2 | 14 | 83.0/27.1 |
| 71 | CAU 1645 | 024320865.1 | *M. grossiae* | 81.4/27.9 | 8 | 80.6/24.1 |
| 72 | SMC-4 | 025263265.1 | *M. duvalii* | 84.4/27.7 | 73 | 82.1/25.7 |
| 73 | SMC-8 | 025263565.1 | *M. vaccae* | 85.7/27.9 | 56 | 83.4/26.3 |
| 74 | CPCC 205710 | 025345615.1 | *M. rutilum* | 81.8/24.5 | 17 | 81.6/24.6 |
| 75 | J2 | 026240945.1 | *M. cosmeticum* | 91.7/44.8 | 84 | 81.8/24.5 |
| 76 | CVI_P3 | 026242035.1 | *M. helvum* | 84.7/28.0 | 59 | 85.2/29.3 |
| 77 | 21IE208 | 026501225.1 | *M. phocaicum* | 91.5/44.9 | 67 | 91.2/38.6 |
| 78 | BiH015 | 027706845.1 | *M. iranicum* | 91.6/45.3 | 21 | 89.3/37.9 |
| 79 | VKM Ac-1816D | 000416385.1 | *M. neoaurum* | 93.0/50.0 | 20 | 82.5/25.1 |
| 80 | 236(2023) | 029623955.1 | *M. iranicum* | 83.6/26.4 | 78 | 83.7/26.3 |
| 81 | OTB74 | 029892685.1 | *M. llatzerense* | 82.3/24.7 | 31 | 82.4/25.6 |
| 82 | AC80 | 029893345.1 | *M. nivoides* | 85.3/28.8 | 55 | 87.4/34.5 |
| 83 | TUM20985 | 030295745.1 | *M. hodleri* | 83.0/28.7 | 45 | 83.5/28.9 |
| 84 | na | 900078775.1 | *M. diernhoferi* | 90.0/39.0 | 12 | 84.4/29.5 |
| 85 | 3519A | 900240945.1 | *M. rutilum* | 81.6/24.3 | 27 | 93.0/50.4 |
| 86 | ANDR5 | 022760805.1 | *M. massilipolynesiensis* | 84.1/29.8 | 57 | 84.1/26.5 |

^a^The strain with genome sequence deposited in GenBank at the earliest date was selected as the reference strain for the newly identified taxa.

Table S3**.** Summary of eight subclades in FVC

| Group Label | Known Species no. | Genomes no. | New Taxa no. | Notable Clinically Associated Taxa |
| --- | --- | --- | --- | --- |
| Group 1 | 9 | 27 | 2 | *M. confluentis* |
| Group 2 | 11 | 33 | 11 | *M. rhodesiae* |
| Group 3 | 7 | 24 | 10 | *M. arabiense* |
| Group 4 | 12 | 75 | 12 | *M. mucogenicum* |
| Group 5 | 22 | 215 | 14 | *M. fortuitum* |
| Group 6 | 6 | 25 | 6 | *M. manitobense* |
| Group 7 | 17 | 70 | 13 | *M. iranicum* |
| Group 8 | 23 | 88 | 18 | *M. flavescens* |

Figure S1. **Additional two phylogenomic tree of 106 type strains and 86 novel taxa within the *Mycobacterium* “*Fortuitum-Vaccae*” clade.** For the upper tree, 1,862 core protein families were identified using the CD-HIT program as described in Gupta et al. 2018 [5]; leveraging these core proteins, the tree was also built with IQ-TREE v2.3.0 [20] under LG model allowing for sites heterogeneity with 1,000 ultra-fast bootstraps, and was visualized and annotated using iTOL v6.9 [21]. The second comprehensive phylogenetic tree was constructed from concatenated sequences for 136 proteins, as detailed in Gupta et al. 2018 [5], which form the established marker set for the phylum *Actinobacteria*. Bar, value indicates the nucleotide substitutions per site. The FVC comprised eight major subclades with 11 to 42 species/taxa in each subclade.


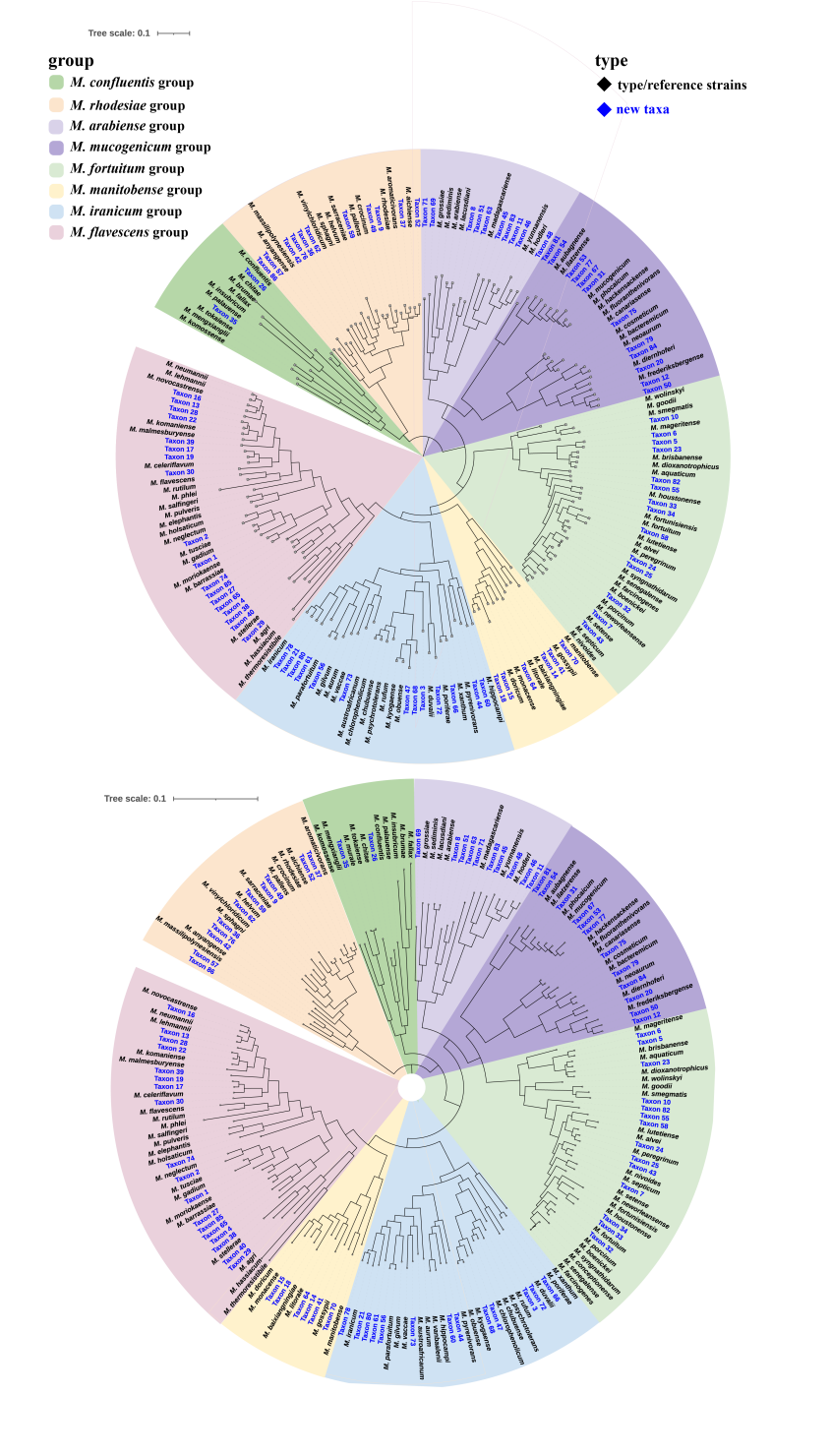


Figure S2. **Taxonomy of 242 genomes with a <95% ANI with all *Mycobacterium* known species.** We examined the taxonomic position of the 242 genomes that had a <95% ANI with all known species. Type or reference strains of all 233 known mycobacterial species were included in building this phylogenetic tree. However, because there are too many genomes, for ease of display, we only show the positions of *M. abscessus*, *M. chelonae*, *M. fortuitum*, *M. vaccae*, *M. triviale*, *M. terrae*, and *M. tuberculosis* as presentatives of known species in this tree. And we found that 119 of the 242 genomes are located within the FVC through this phylogenomic tree. Abbreviations: ANI, average nucleotide identity; FVC, the "*Fortuitum-Vaccae*" clade;


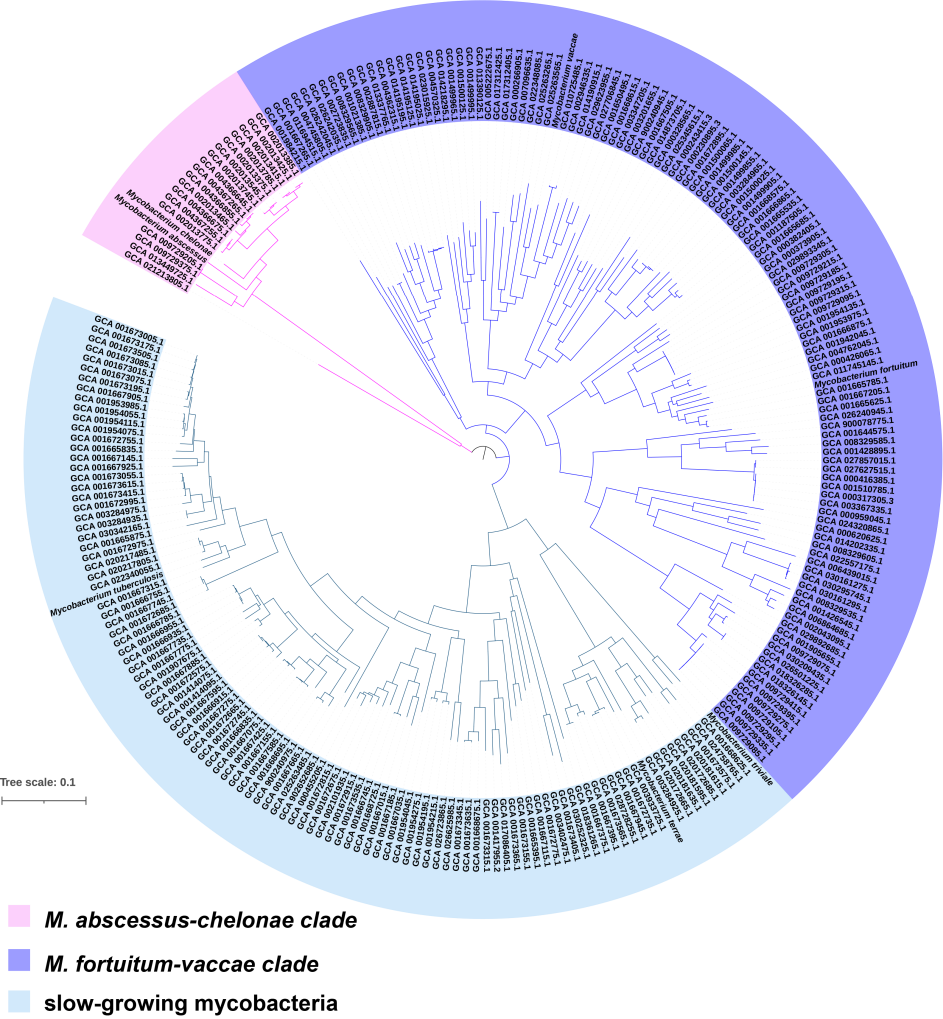

Supplement: Supplementary file 2 [file Table_1.docx]
